# Double lung transplantation is better than single lung transplantation for end-stage chronic obstructive pulmonary disease: a meta-analysis

**DOI:** 10.1186/s13019-024-02654-6

**Published:** 2024-03-30

**Authors:** Yu-Chi Fang, Wen-Hsin Cheng, Hung-I Lu, Yi-Shi Wang, Kai-Hao Chuang, Hsing-Hua Lai, Yu Chen, Li-Chun Chen, Meng-Yun Tsai, Yu-Ping Chang, Kuo-Tung Huang, Chien-Ming Lo

**Affiliations:** 1 Department of Thoracic & Cardiovascular Surgery, Kaohsiung Chang Gung Memorial Hospital, No. 123 Dapi Road, Niaosong Dist., Kaohsiung, Taiwan, ROC; 2 Department of Chest, Kaohsiung Chang Gung Memorial Hospital, Kaohsiung, Taiwan

**Keywords:** Pulmonary disease, Chronic obstructive, Lung transplantation, Proportional hazards models, Registries, Survival, Meta-analysis

## Abstract

**Background:**

Lung transplantation is one of the most common treatment options for patients with end-stage chronic obstructive pulmonary disease. However, the choice between single and double lung transplantation for these patients remains a matter of debate. Therefore, we performed a systematic search of medical databases for studies on single lung transplantation, double lung transplantation, and chronic obstructive pulmonary disease.

**Methods:**

The rate ratio and hazard ratio of survival were analyzed. The meta-analysis included 15 case–control and retrospective registry studies.

**Results:**

The rate ratios of the 3-year survival (0.937 and *P* = 0.041) and 5-year survival (0.775 and *P* = 0.000) were lower for single lung transplantation than for double lung transplantation. However, the hazard ratio did not differ significantly between the two.

**Conclusions:**

Double lung transplantation was found to provide better benefits than single lung transplantation in terms of the long-term survival in patients with chronic obstructive pulmonary disease.

**Supplementary Information:**

The online version contains supplementary material available at 10.1186/s13019-024-02654-6.

## Background

Chronic obstructive pulmonary disease (COPD) is the most common indication for lung transplantation worldwide ^1^. Currently, lung transplantation is the final treatment strategy for patients with end-stage COPD. The points in favor of single lung transplantation (SLT) and double lung transplantation (DLT) are equivocal. However, researchers of some case–control series have reported better outcomes in patients who underwent DLT than in those who underwent SLT [1]; in their experience, SLT leads to a high rate of primary graft dysfunction. Conversely, a large retrospective registry analysis revealed equal outcomes between SLT and DLT [2]. We reviewed relevant published literature and noted two different opinions regarding SLT and BLT: most studies have indicated that DLT is better for survival than SLT, but others have provided data indicating equal outcomes between the two.

Therefore, we reviewed the existing literature on the subject and performed a meta-analysis of all included studies to determine whether SLT or DLT yielded better survival outcomes.

## Methods

### Search strategy and inclusion criteria

We searched the PubMed, Medline, and Scopus databases using one or more of the following keywords: “chronic obstructive pulmonary disease” and “single lung transplantation or double lung transplantation.” A total of 416 results were identified in the search. We excluded articles on animal studies; articles written in a language other than English; articles that were case reports, reviews, letters, and editorial comments; articles published before 2000; and articles on studies with less than 50 patients.

The primary inclusion criteria were that the study must compare two treatment arms, (i.e., SLT and DLT) and that all the included patients should have undergone lung transplantation for end-stage lung disease.

### Data extraction and quality assessment

Two reviewers read all the included literature critically and extracted the relevant data, including the first author, year of publication, number of treatment arms, and survival results. The quality of the included studies was assessed by all authors using the Newcastle–Ottawa Scale, which comprises three parts for a case–control study or cohort study: “SELECTION” (four items), “COMPARABILITY” (one item), and “EXPOSURE” (three items). Disagreements between the two reviewers were resolved through discussions with the other authors, including the corresponding author.

### Data synthesis and analysis

Patient survival was the primary outcome in this study. We used rate ratios to compare SLT and DLT. Some of the included studies used multiple variance analyses and presented data with hazard ratios; we also used these to compare SLT and DLT. A random effects model was used to pool individual rate ratios and hazard ratios. Heterogeneity was determined using I^2^ tests; I^2^ values of > 50% were considered indicative of obvious heterogeneity. Potential publication bias was determined using the Egger’s test and Funnel plots. Statistical significance was defined as *P* < 0.05. All statistical analyses were performed using the Comprehensive Meta-Analysis software, version 3 (Biostat, Englewood, NJ, USA).

## Results

### Study search and characteristics of the included patients

**Fig. 1:**
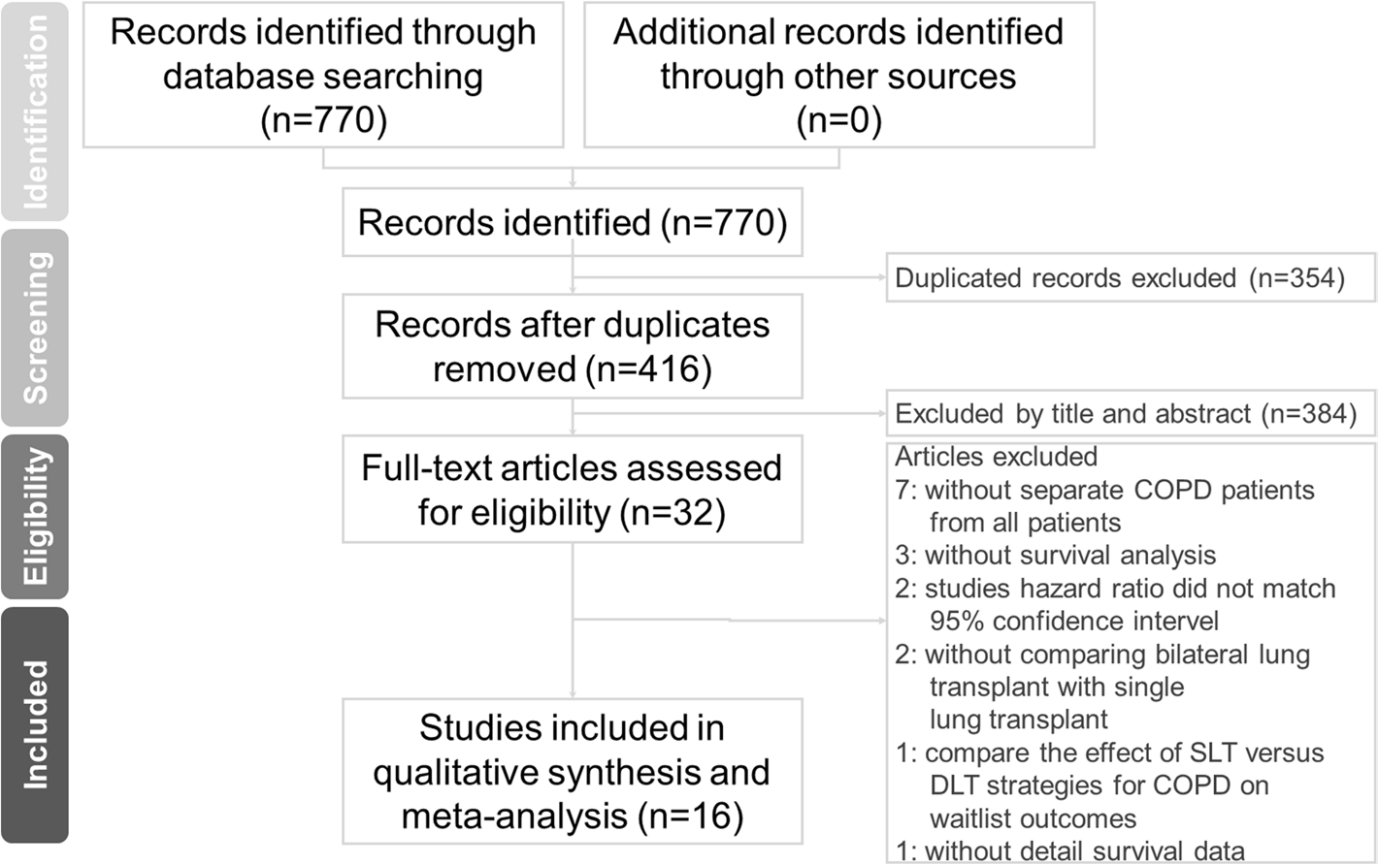
Preferred reporting items for systematic reviews and meta-analyses (PRISMA) flow diagram

Overall, 416 records were identified through database searching. Two reviewers read the titles, abstracts, and keywords of these records, and selected 32 studies based on the inclusion and exclusion criteria (Fig. 1). These mostly comprised case–control studies and database analyses.

Seventeen of these were further excluded for the following reasons: patients with COPD were not separated from all lung transplant recipients [3–10]; survival data were not analyzed [11–13]; problems were noted with the statistical analyses, i.e., hazard ratios did not fit the 95% confidence intervals [14, 15]; SLT and DLT were not compared [16, 17]; a simulated model was used to compare the effects of SLT and DLT for COPD on waitlist outcomes, but long-term survival data were not reported [18]; and the OPTN/UNOS database was analyzed, but detailed survival data were not reported [19].

Finally, 15 studies remained for data analysis; most comprised retrospective case–control studies [1, 2, 20–32]. Some of these were single-center, retrospective case–control studies [1, 21, 23, 24, 28, 29]; the others were database cohort analyses [2, 20, 22, 25–27, 30–32]. The data extracted from all the included studies are provided in Table 1. We used the Newcastle–Ottawa Scale to appraise all the studies; the results are provided in Table 2.

**Table 1:** Patient characteristics, study methodology, and quality assessment of included trial

Author, year	Patients’ Diagnosis	Surgery	Study Design	Enrolled sample number	Mean age, years	Outcome measurement	Quality assessment
Pochettino et al., 2000 [1]	COPD	Lung Transplant	Retrospective Case–control	DLT: 46 SLT: 84	DLT: 51.1 ± 1.2 SLT: 56.2 ± 0.7	Functional assessment, overall survival, and survival following onset of BOS	7
Meyer et al., 2001 [20]	Emphysema/COPD	Lung Transplant	Retrospective cohort (ISHLT/UNOS database)	DLT: 425 SLT: 1835	DLT: 50.5 ± 7.1 SLT: 54.8 ± 6.3	Mortality and Morbidity analysis	8
Cassivi et al., 2002 [21]	COPD/AATD	Lung Transplant	Retrospective Case–control	DLT: 112 SLT: 228	Total: 55.2 ± 6.4	Survival, Posttransplant functional result, morbidity	7
Burton et al., 2005 [22]	All suitable for lung transplant cases	Lung Transplant	Retrospective Case–control	DLT: 228 SLT: 112	(Median) DLT: 45 SLT: 56	Survival rates for the center as a whole and compared survival rates between different sub-groups of patients	7
Hadjiliadis et al., 2006 [24]	COPD/AATD	Lung Transplant	Retrospective cohort (2 centers)	DLT: 103 SLT: 118	DLT: 53.0 ± 7.8 SLT: 55.3 ± 8.0	The effect of transplant on the development of BOS, survival, and survival after BOS in patients with COPD and alpha-1-antitrypsin deficiency	7
Gunes et al., 2006 [23]	COPD	Lung Transplant	Retrospective Case–control	DLT: 99 SLT: 66	Total: 50 ± 6	Outcomes of lung transplant for COPD (A-1 & cigarette smoking)	7
Stavem et al., 2006 [25]	All suitable for lung transplant cases	Lung Transplant	Retrospective Case–control	DLT: 37 SLT: 49	Total: 49.6 ± 8.9	The impact of diagnosis, SLT vs BLT, and the timing of transplant on the survival of patients placed on the waiting list for lung transplantation in Norway from 1990 to 2003	7
Nwakanma et al., 2007 [26]	All suitable for lung transplant cases over 60 years old	Lung Transplant	Retrospective Cohort (UNOS)	DLT: 224 SLT: 863	(All Diagnosis) DLT: 62.3 ± 2.1 SLT: 62.8 ± 2.5	The impact of procedure type on short- and mid-term survival in recipients 60 years of age or older for all disease types	7
Thabut et al., 2008 (Am J Respir Crit Care Med) [27]	COPD	Lung Transplant	Retrospective Cohort (UNOS)	Total: 5873 DLT: 27.8% SLT: 72.2%	Total: 56.0 ± 7.1	Analyze data from the UNOS registry to (1) determine the survival (2) patients with COPD most likely to benefit from LT (3) create an instrument for caregivers to compute the expected survival effect of LT for a given patient	7
Thabut et al., 2008 (Lancet) [2]	COPD	Lung Transplant	Retrospective Cohort (ISHLT)	DLT: 3525 SLT: 6358	DLT: 52.2 ± 7.8 SLT: 55.5 ± 6.8	Compare survival rates after bilateral and single lung transplantation for patients with COPD	8
Delgado et al., 2009 [28]	Pulmonary emphysema	Lung Transplant	Retrospective Case–control	DLT: 33 SLT: 29	Total: 53.89 ± 6.75	Propose single lung transplant as the first-choice treatment for patients with a diagnosis of emphysema	6
Bennett et al., 2015 [29]	COPD	Lung Transplant	Retrospective Case–control (Single-Center) and Cohort (UNOS)	DLT: 30 SLT: 206 (UNOS) DLT: 2848	DLT: 58.89 ± 4.54 SLT: 52.41 ± 5.02 (UNOS) DLT: 57.48 ± 7.47	Comparable 5-year survival among the single-center SLTx cohort and BLTx recipients at both the local and national levels	8
Schaffer et al., 2015 [30]	COPD/IPF	Lung Transplant	Retrospective Cohort (ISHLT)	DLT: 1875 SLT: 1299	DLT: 59.4 ± 6.4 SLT: 61.6 ± 5.5	Reviewed UNOS data to summarize outcomes of IPF and COPD who underwent single- or double lung transplantation since the LAS was implemented	8
Gulack et al., 2018 [31]	COPD/AATD	Lung Transplant	Retrospective Cohort (UNOS)	DLT: 5688 SLT: 3881	(Median) DLT: 57 SLT: 58	Hypothesized that patients with AATD have superior long-term survival following lung transplantation than patients with COPD	8
Crawford et al., 2019 [32]	COPD	Lung Transplant	Retrospective Cohort (UNOS)	DLT: 2196 SLT: 1358	DLT: 60 ± 6 SLT: 62 ± 5	Evaluate survivals in lung transplant recipients with COPD among SLT and DLT cohorts	8

*Abbreviations*: *AATD* alpha-1-antitrypsin deficiency, *DLT* double lung transplant, *SLT* single lung transplant, *COPD* chronic obstructive pulmonary disease, *IPF* idiopathic pulmonary fibrosis, *BOS* bronchiolitis obliterans syndrome, *UNOS* United Network for Organ Sharing, *ISHLT* International Society for Heart and Lung Transplantation

**Table 2:** The detail of quality assessment of the included studies

Case–control Study									
Author, year	Selection				Comparability	Exposure			Quality assessment
	Is the case definition adequate?	Representativeness of the cases	Selection of Controls	Definition of Controls	Comparability of cases and controls on the basis of the design or analysis	Ascertainment of exposure	Same method of ascertainment for cases and controls	Non-response rate	
Pochettino et al., 2000 [1]	*	*		*	**	*	*		7
Cassivi et al., 2002 [21]	*	*		*	**	*	*		7
Burton et al., 2005 [22]	*	*		*	**	*	*		7
Gunes et al., 2006 [23]	*	*		*	**	*	*		7
Stavem et al., 2006 [25]	*	*		*	**	*	*		7
Delgado et al., 2009 [28]	*	*		*	*	*	*		6
	Selection				Comparability	Exposure			Quality assessment
	Representativeness of the exposed cohort	Selection of the non-exposed cohort	Ascertainment of exposure	Demonstration that outcome of interest was not present at the start of study	Comparability of cohorts on the basis of the design or analysis	Assessment of outcome	Was follow-up long enough for outcomes to occur?	Adequacy of follow-up of cohorts	
Meyer et al., 2001 [20]	*	*		*	**	*	*	*	8
Hadjiliadis et al., 2006 [24]	*	*		*	**	*	*	*	8
Nwakanma et al., 2007 [26]	*	*		*	**	*	*		7
Thabut et al., 2008 (Am J Respir Crit Care Med) [27]	*	*		*	**	*	*		7
Thabut et al., 2008 (Lancet) [2]	*	*		*	**	*	*	*	8
Bennett et al., 2015 [29]	*	*		*	**	*	*	*	8
Schaffer et al., 2015 [30]	*	*		*	**	*	*	*	8
Gulack et al., 2018 [31]	*	*		*	**	*	*	*	8

### Pooled rate ratio and hazard ratio of survival

We analyzed the survival rate and compared the same between the SLT and DLT groups in each study. We also included the 1-year, 3-year, and 5-year survival data in the analysis. In some studies, results were obtained using multiple variance analyses and hazard ratios; we performed a separate analysis for these studies [2, 25, 27, 30].

**Fig. 2:**
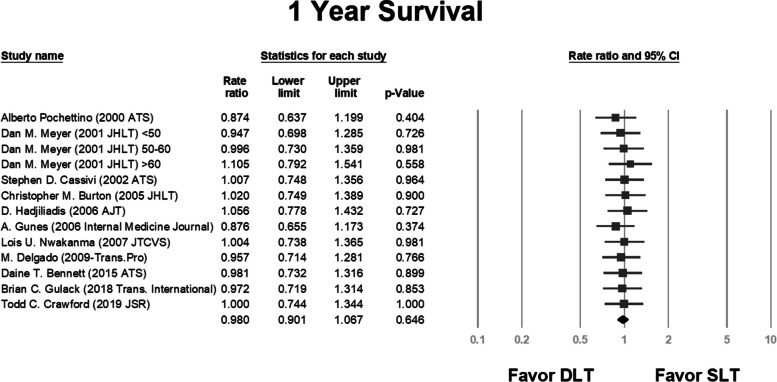
Rate ratio analysis of 1-year survival following double and single lung transplantation. CI, confidence interval; DLT, double lung transplantation; SLT, single lung transplantation

**Fig. 3:**
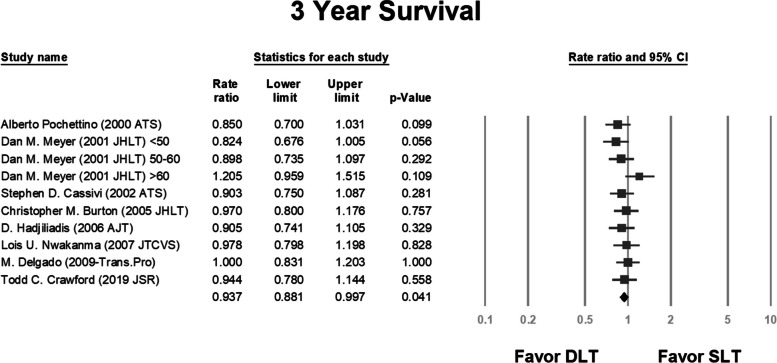
Rate ratio analysis of 3-year survival following double and single lung transplantation. CI, confidence interval; DLT, double lung transplantation; SLT, single lung transplantation

**Fig. 4:**
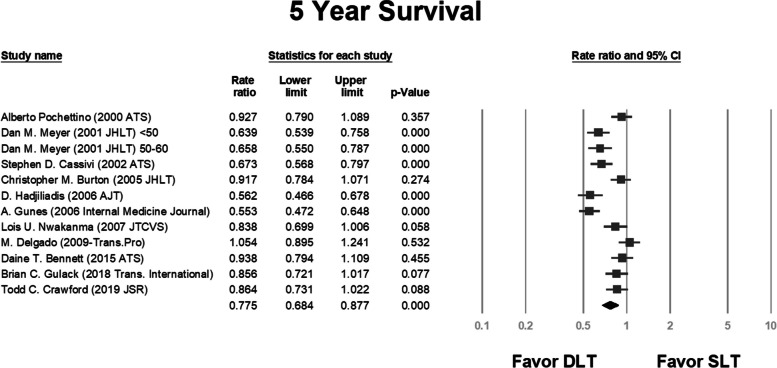
Rate ratio analysis of 5-year survival following double and single lung transplantation. CI, confidence interval; DLT, double lung transplantation; SLT, single lung transplantation

The pooled rate ratios were 0.98 (*P* = 0.646; Fig. 2), 0.937 (*P* = 0.041; Fig. 3), and 0.775 (*P* = 0.000; Fig. 4) for the 1-year, 3-year, and 5-year survival, respectively.

**Fig. 5:**
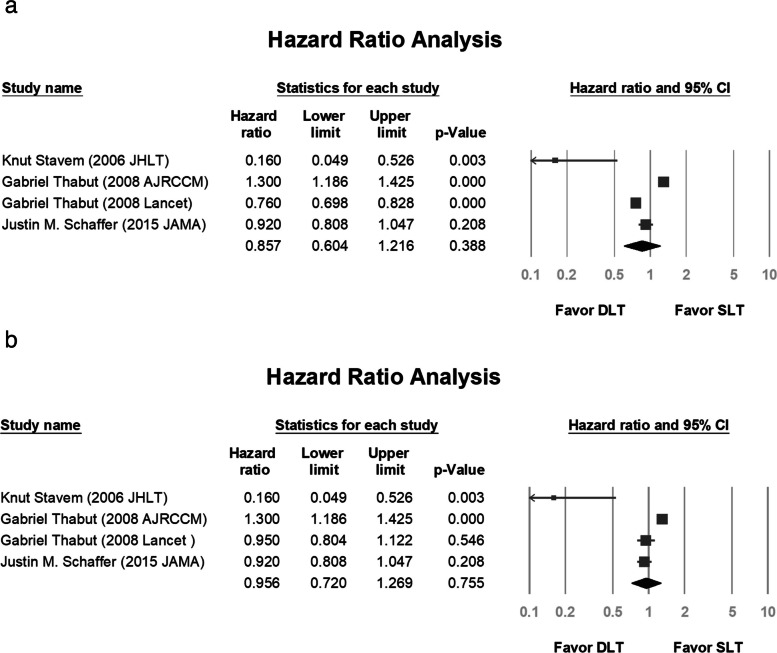
Hazard ratio analysis of double and single lung transplantation. **a** Without propensity score matching in the study by Thabut et al. **b** With propensity score matching. CI, confidence interval; DLT, double lung transplantation; SLT, single lung transplantation

The pooled hazard ratio of survival was 0.857 (*P* = 0.388; Fig. 5a). Thabut et al. analyzed the International Society for Heart and Lung Transplantation database and reported different data after propensity score matching [2]. We included their study, with two different results, in the analysis because the *P* values were not significant. The pooled hazard ratio was 0.956 (*P* = 0.755; Fig. 5b).

**Fig. 6:**
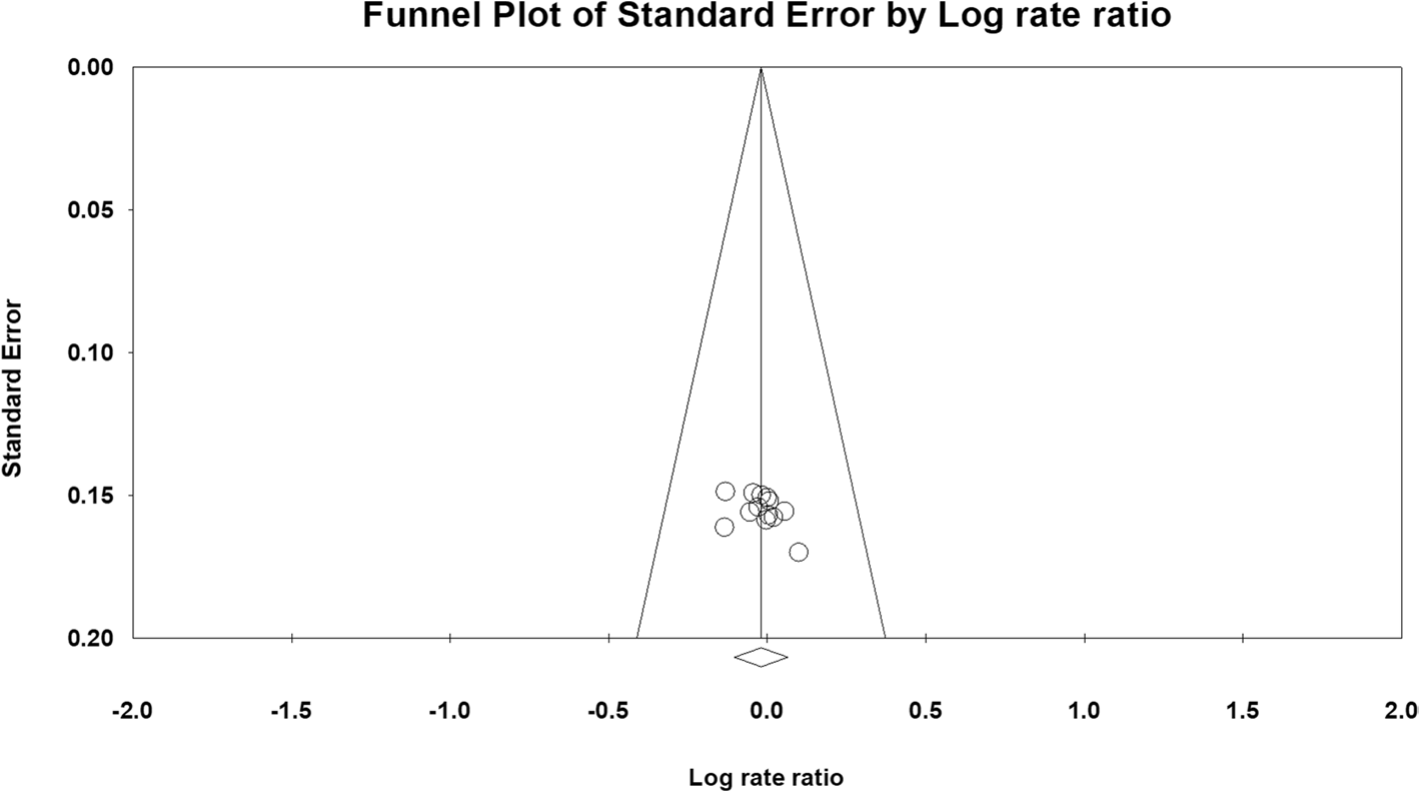
Funnel plot of all studies that included 1-year survival data

**Fig. 7:**
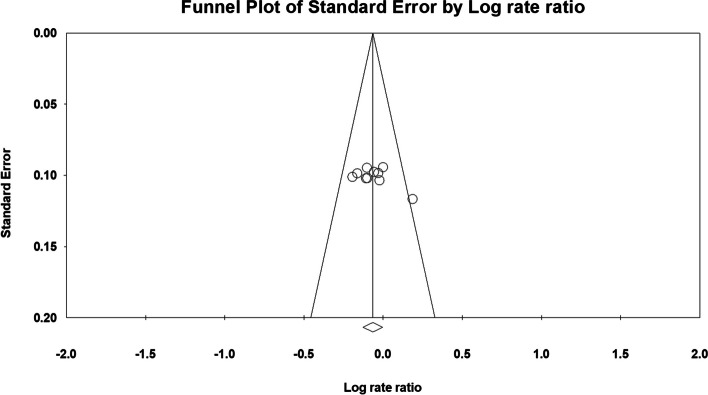
Funnel plot of all studies that included 3-year survival data

**Fig. 8:**
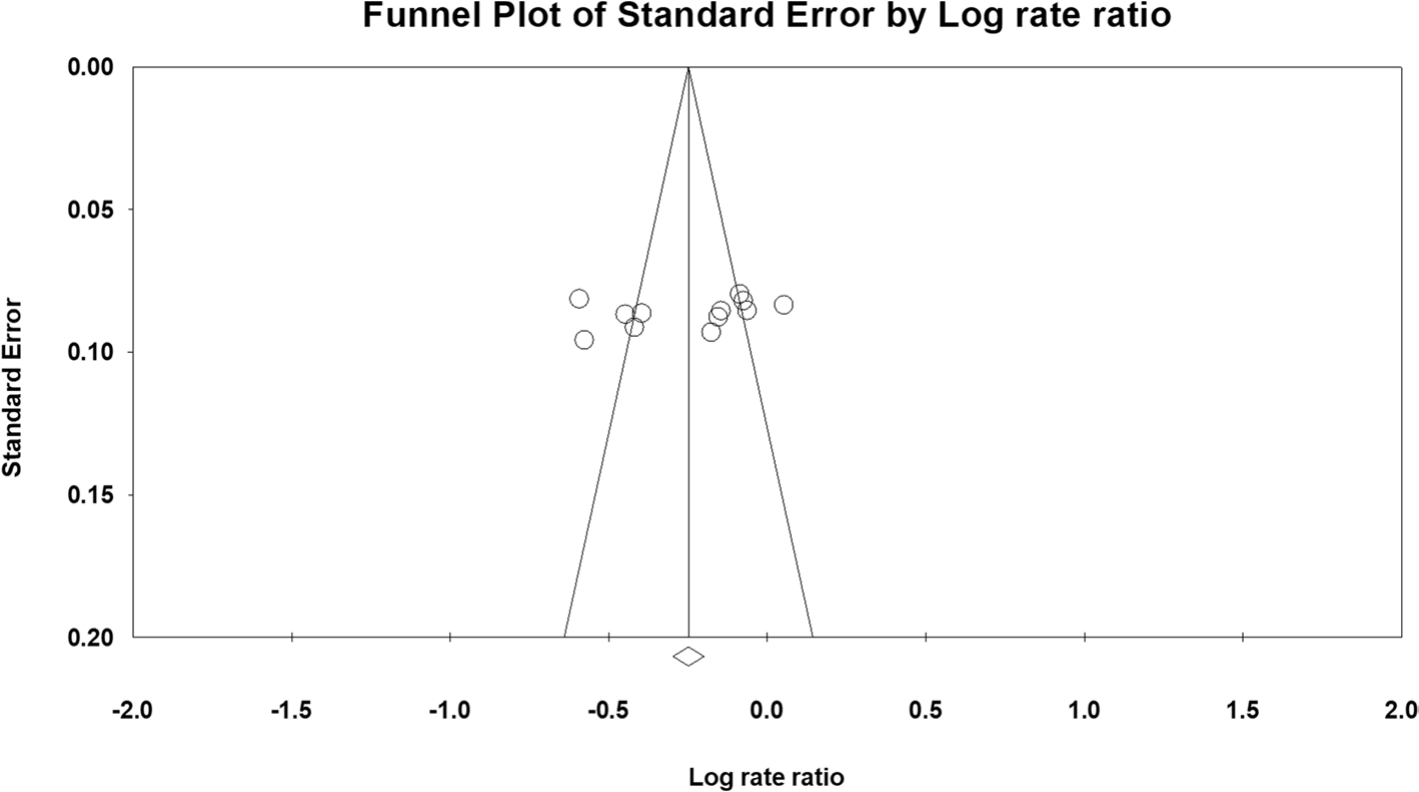
Funnel plot of all studies that included 5-year survival data

**Fig. 9:**
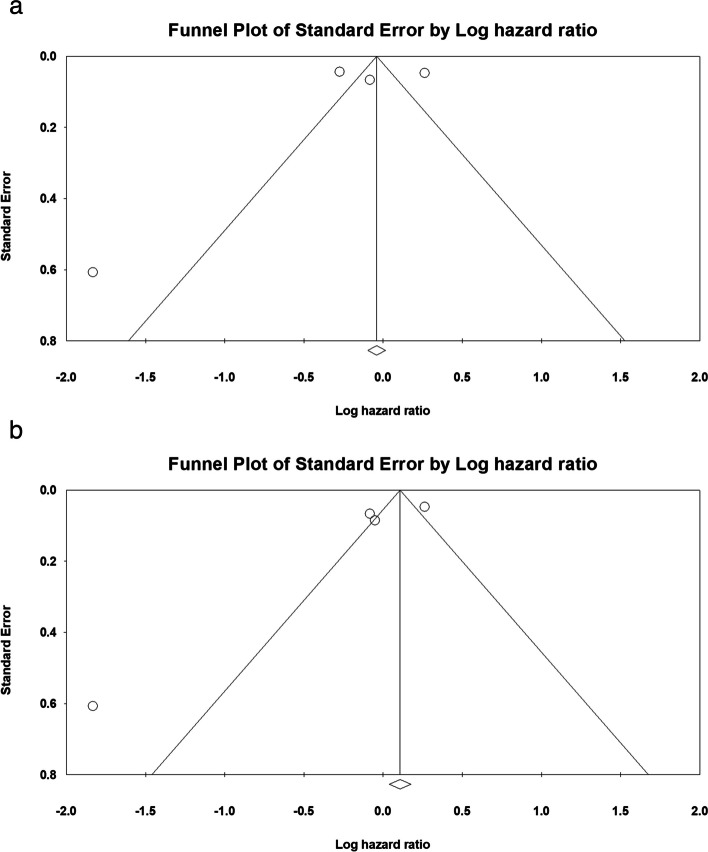
Funnel plot of all studies included in the hazard ratio analysis. **a** Without propensity score matching in the study by Thabut et al. **b** With propensity score matching

The Egger’s test did not reveal a significant publication bias in the following: 1) pooled rate ratio analyses of the 1-year (*P* = 0.154), 3-year (*P* = 0.097), and 5-year (*P* = 0.242) survival; 2) hazard ratio analysis (*P* = 0.711); and 3) hazard ratio analysis with Thabut et al.’s propensity score matching results (*P* = 0.188). The Funnel plots are presented in Figs. 6, 7, 8, 9a and b.

## Discussion

Patients with end-stage COPD are often recommended to undergo lung transplantation, which is currently the most acceptable treatment method. However, the debate between SLT and DLT still exists [33]. Lung transplantation is a complex and difficult surgery, and procedure selection is known to affect patient survival [34].

Our analysis showed that the early survival outcomes were equal between SLT and DLT. However, DLT achieved a better mid-term and long-term survival than SLT. The pooled hazard ratio did not reveal a significant difference between the two.

Most of the case–control series revealed a better outcome for DLT [1, 21, 24, 28, 29]. However, analysis studies based on a large registry revealed equal outcomes for both methods [2, 30]. Besides, DLT could bring about an organ shortage and increase the risk of mortality in patients on the waiting list. This is the primary reason the current review did not recommend whether patients with end-stage COPD should receive SLT or DLT.

The retrospective database study by Thabut et al. is an important one; it majorly contributed to the present meta-analysis due to its large sample size. Thabut et al. used different statistical methods (including propensity score matching) in an attempt to reduce the effect of confounding factors. They achieved the same result with these methods. We chose to include their study because we thought that their data, obtained with multiple methods, would allow us to better compare SLT and DLT.

The choice between DLT and SLT remains debatable. Waiting list mortality is major concern during choosing the appropriate procedure. SLT can reduce the waiting times associated with organ shortage [29]. However, DLT has been proven to yield better survival and quality of life outcomes in some studies [1]. This conflict will affect the choice of procedure, especially when the patient’s age is taken into consideration. DLT could provide a better quality of life for larger lung volumes [1]. For younger recipients, this is an important factor to consider while discussing the treatment plans with the transplantation team.

Our study had several limitations. First, all the included studies were case–control studies or retrospective analyses of registry data. Thus, the evidence level was not high. Several additional factors affect patient survival, including the patient’s age, center where the surgery is conducted and the facilities available there, and the surgeon’s experience and expertise. Two of the included studies involved age-based analyses [2, 20]; however, the meta-analysis pooled their data and masked the effect of age.

Furthermore, we excluded studies published before 2000 because surgery techniques and critical care have undergone significant changes in the past 20 years. The aforementioned factors would have affected our results had we included studies published before 2000 in our meta-analysis. Accordingly, we further excluded case–control studies with less than 50 patients since such low-volume studies could also affect our results.

It is impossible to conduct a prospective randomized trial on this subject due to ethical considerations regarding patient treatment. However, a retrospective registry analysis across multiple countries and comparison of the obtained results may provide data beneficial for patients with end-stage COPD worldwide. The more retrospective studies published, the more data we can collect for a meta-analysis to determine the different factors related to the outcomes of the two transplantation procedures.

## Conclusions

We determined that in patients with end-stage COPD, DLT results in a better 3-year and 5-year survival than SLT.

### Supplementary Information

**Additional file 1. **Preoperative demographic characteristics; Operative demographics; Postoperative demographics. Collect some factors from inclusive studies.

## Data Availability

The data that support the findings of this study are openly available in PubMed, Medline, and Scopus.

## Abbreviations

COPD: Chronic obstructive pulmonary disease
DLT: Double lung transplantation
SLT: Single lung transplantation
