# Supplementary material for: Double lung transplantation is better than single lung transplantation for end-stage chronic obstructive pulmonary disease: a meta-analysis
Source: J Cardiothorac Surg. 2024 Mar 30;19:162. doi: 10.1186/s13019-024-02654-6 (PMC10981328; doi:10.1186/s13019-024-02654-6)
Supplement: Supplementary file 1 — Additional file 1. Preoperative demographic characteristics; Operative demographics; Postoperative demographics. Collect some factors from inclusive studies. [file 13019_2024_2654_MOESM1_ESM.docx]

| Supplement Table 1: Preoperative Demographics | | | | | | | | |
| --- | --- | --- | --- | --- | --- | --- | --- | --- |
|  | Ages (years) | | Sex (% male) | | FEV1 (%predicted) | | 6MWT(ft) | |
|  | SLT | BLT | SLT | BLT | SLT | BLT | SLT | BLT |
| Alberto Pochettino et.al ^1^ | 56.2± 0.7 | 51.1± 1.2 | 46 | 66 | 19.8± 0.8 | 19.0± 0.9 | 867± 34 | 951± 43 |
| Dan M. Meyer, et.al ^2^ | 54.8± 6.3 | 50.5± 7.1 | 46 | 57 | NA | NA | NA | NA |
| Stephen D. Cassivi et.al ^3^ | NA | NA | NA | NA | NA | NA | NA | NA |
| Christopher M.Burton et.al^4^ | 56 | 45 | 41 | 53 | NA | NA | NA | NA |
| D. Hadjiliadis et.al^5^ | 53.0±7.8 | 55.3±8.0 | 42 | 56 | NA | NA | NA | NA |
| A.Gunes et.al^6^ | 50.5±5.3 | 50±5.9 | 67 | 47 | NA | NA | 722±354 | 988±433 |
| Lois U. Nwakanma et.al^7^ | 62.8±2.5 | 62.3±2.1 | 57 | 63 | 34.5±20.6 | 35.5±23.4 | NA | NA |
| M. Delgado et.al ^8^ | NA | NA | NA | NA | NA | NA | NA | NA |
| Daine T.Bennett et.al ^9^ | 52.41±5.02 | 58.89±4.54 | 51.5 | 50 | 18.13±6.1 | 19.93±6.84 | 609.63±255.52 | 640.35±272.3 |
| Brian C.Gulack et.al ^10^ | 58 | 57 | 47 | 55 | 20 | 19 | NA | NA |
| Todd C. Crawford et.al ^11^ | 62±5 | 60±6 | 48 | 55 | NA | NA | NA | NA |
| Knut Stavem et.al ^12^ | 51.4 | 49.8 | 66 | 70 | 25 | 23 | 853 | 1026 |
| Gabriel Thabut et.al ^13^ | NA | NA | NA | NA | NA | NA | NA | NA |
| Gabriel Thabut et.al ^14^ | 55.5 | 52.2 | 47 | 56 | 22.2 | 21.7 | NA | NA |
| Justin M.Schaffer et.al ^15^ | 61.6 | 59.4 | 47 | 55 | NA | NA | 715 | 752 |

**Supplement Data Tables:**

| Supplement Table 2: Operative Demographics | | | | | | |
| --- | --- | --- | --- | --- | --- | --- |
|  | Preop ECLS(%) | | ECLS in operation(%) | | Postop ECLS(%) | |
|  | SLT | BLT | SLT | BLT | SLT | BLT |
| Alberto Pochettino et.al ^1^ | NA | NA | NA | NA | NA | NA |
| Dan M. Meyer, et.al ^2^ | 1.7 | 2.3 | NA | NA | NA | NA |
| Stephen D. Cassivi et.al ^3^ | NA | NA | NA | NA | NA | NA |
| Christopher M.Burton et.al^4^ | NA | NA | NA | NA | NA | NA |
| D. Hadjiliadis et.al^5^ | NA | NA | NA | NA | NA | NA |
| A.Gunes et.al^6^ | NA | NA | NA | NA | NA | NA |
| Lois U. Nwakanma et.al^7^ | NA | NA | NA | NA | NA | NA |
| M. Delgado et.al ^8^ | NA | NA | NA | NA | NA | NA |
| Daine T.Bennett et.al ^9^ | NA | NA | NA | NA | NA | NA |
| Brian C.Gulack et.al ^10^ | NA | NA | NA | NA | NA | NA |
| Todd C. Crawford et.al ^11^ | 0 | 0.5 | NA | NA | NA | NA |
| Knut Stavem et.al ^12^ | NA | NA | NA | NA | NA | NA |
| Gabriel Thabut et.al ^13^ | NA | NA | NA | NA | NA | NA |
| Gabriel Thabut et.al ^14^ | NA | NA | NA | NA | NA | NA |
| Justin M.Schaffer et.al ^15^ | 0 | 0.5 | NA | NA | NA | NA |

| Supplement Table 3: Postoperative Demographics | | | | | | | | |
| --- | --- | --- | --- | --- | --- | --- | --- | --- |
|  | 90 Day Mortality (%) | | Mean Follow up time (months) | | FEV1(1 year after LTx)(%predicted) | | 6MWT(ft)(1 year after LTx) | |
|  | SLT | BLT | SLT | BLT | SLT | BLT | SLT | BLT |
| Alberto Pochettino et.al ^1^ | 15.5 | 13.0 | 32.4 | 32.4 | 50 | 80 | 1200 | 1600 |
| Dan M. Meyer, et.al ^2^ | 11 | 11 | NA | NA | NA | NA | NA | NA |
| Stephen D. Cassivi et.al ^3^ | 7.0 | 5.9 | NA | NA | NA | NA | NA | NA |
| Christopher M.Burton et.al^4^ | 9.7 | 10.8 | NA | NA | NA | NA | NA | NA |
| D. Hadjiliadis et.al^5^ | 11.3 | 12.7 | 34.5 | 44.6 | NA | NA | NA | NA |
| A.Gunes et.al^6^ | NA | NA | 56 | 56 | NA | NA | NA | NA |
| Lois U. Nwakanma et.al^7^ | 4.8 | 5.1 | NA | NA | NA | NA | NA | NA |
| M. Delgado et.al ^8^ | NA | NA | NA | NA | NA | NA | NA | NA |
| Daine T.Bennett et.al ^9^ | 3.3(30days) | 3.7(30days) | NA | NA | NA | NA | NA | NA |
| Brian C.Gulack et.al ^10^ | NA | NA | NA | NA | NA | NA | NA | NA |
| Todd C. Crawford et.al ^11^ | NA | NA | 37 | 37 | NA | NA | NA | NA |
| Knut Stavem et.al ^12^ | NA | NA | NA | NA | NA | NA | NA | NA |
| Gabriel Thabut et.al ^13^ | NA | NA | NA | NA | NA | NA | NA | NA |
| Gabriel Thabut et.al ^14^ | NA | NA | 80 | 80 | NA | NA | NA | NA |
| Justin M.Schaffer et.al ^15^ | NA | NA | NA | NA | NA | NA | NA | NA |

Reference:

1. Pochettino A, Kotloff RM, Rosengard BR, et al. Bilateral versus single lung transplantation for chronic obstructive pulmonary disease: intermediate-term results. *The Annals of thoracic surgery.* 2000;70(6): 1813-1818; discussion 1818-1819.

2. Meyer DM, Bennett LE, Novick RJ, Hosenpud JD. Single vs bilateral, sequential lung transplantation for end-stage emphysema: influence of recipient age on survival and secondary end-points. *The Journal of heart and lung transplantation : the official publication of the International Society for Heart Transplantation.* 2001;20(9): 935-941.

3. Cassivi SD, Meyers BF, Battafarano RJ, et al. Thirteen-year experience in lung transplantation for emphysema. *The Annals of thoracic surgery.* 2002;74(5): 1663-1669; discussion 1669-1670.

4. Burton CM, Milman N, Carlsen J, et al. The Copenhagen National Lung Transplant Group: survival after single lung, double lung, and heart-lung transplantation. *The Journal of heart and lung transplantation : the official publication of the International Society for Heart Transplantation.* 2005;24(11): 1834-1843.

5. Hadjiliadis D, Chaparro C, Gutierrez C, et al. Impact of lung transplant operation on bronchiolitis obliterans syndrome in patients with chronic obstructive pulmonary disease. *American journal of transplantation : official journal of the American Society of Transplantation and the American Society of Transplant Surgeons.* 2006;6(1): 183-189.

6. Güneş A, Aboyoun CL, Morton JM, Plit M, Malouf MA, Glanville AR. Lung transplantation for chronic obstructive pulmonary disease at St Vincent's Hospital. *Internal medicine journal.* 2006;36(1): 5-11.

7. Nwakanma LU, Simpkins CE, Williams JA, et al. Impact of bilateral versus single lung transplantation on survival in recipients 60 years of age and older: analysis of United Network for Organ Sharing database. *The Journal of thoracic and cardiovascular surgery.* 2007;133(2): 541-547.

8. Delgado M, Borro JM, De La Torre MM, et al. Lung transplantation as the first choice in emphysema. *Transplantation proceedings.* 2009;41(6): 2207-2209.

9. Bennett DT, Zamora M, Reece TB, et al. Continued Utility of Single-Lung Transplantation in Select Populations: Chronic Obstructive Pulmonary Disease. *The Annals of thoracic surgery.* 2015;100(2): 437-442.

10. Gulack BC, Mulvihill MS, Ganapathi AM, et al. Survival after lung transplantation in recipients with alpha-1-antitrypsin deficiency compared to other forms of chronic obstructive pulmonary disease: a national cohort study. *Transplant international : official journal of the European Society for Organ Transplantation.* 2018;31(1): 45-55.

11. Crawford TC, Lui C, Magruder JT, et al. Five-Year Mortality Hazard Is Reduced In Chronic Obstructive Pulmonary Disease Patients Receiving Double- versus Single-Lung Transplants. *The Journal of surgical research.* 2019;237: 118-125.

12. Stavem K, Bjørtuft Ø, Borgan Ø, Geiran O, Boe J. Lung transplantation in patients with chronic obstructive pulmonary disease in a national cohort is without obvious survival benefit. *The Journal of heart and lung transplantation : the official publication of the International Society for Heart Transplantation.* 2006;25(1): 75-84.

13. Thabut G, Ravaud P, Christie JD, et al. Determinants of the survival benefit of lung transplantation in patients with chronic obstructive pulmonary disease. *American journal of respiratory and critical care medicine.* 2008;177(10): 1156-1163.

14. Thabut G, Christie JD, Ravaud P, et al. Survival after bilateral versus single lung transplantation for patients with chronic obstructive pulmonary disease: a retrospective analysis of registry data. *Lancet (London, England).* 2008;371(9614): 744-751.

15. Schaffer JM, Singh SK, Reitz BA, Zamanian RT, Mallidi HR. Single- vs double-lung transplantation in patients with chronic obstructive pulmonary disease and idiopathic pulmonary fibrosis since the implementation of lung allocation based on medical need. *Jama.* 2015;313(9): 936-948.
